# Smoke-Free Multiunit Housing Policy: Caretakers’ Perspectives on Economic and Personal Impacts

**DOI:** 10.3390/ijerph120708092

**Published:** 2015-07-15

**Authors:** Rachel E. Wilbur, Anna H. Stein, Elena M. Pinzon, Osub S. Ahmed, Obie S. McNair, Kurt M. Ribisl

**Affiliations:** 1 Gillings School of Global Public Health, University of North Carolina, Chapel Hill, NC 27599, USA; E-Mails: rachel.e.wilbur@gmail.com (R.E.W.); epinzon28@gmail.com (E.M.P.); osubahmed@gmail.com (O.S.A.); obie.s.mcnair@gmail.com (O.S.M.); 2 North Carolina Division of Public Health, Raleigh, NC 27699, USA; E-Mail: Anna.Stein@dhhs.nc.gov; 3 Lineberger Comprehensive Cancer Center, University of North Carolina, Chapel Hill, NC 27599, USA

**Keywords:** multiunit housing, smoke-free policy, secondhand smoke, thirdhand smoke, tobacco control policy

## Abstract

Objective: Multiunit housing (MUH) operators may be motivated to adopt smoke-free policies to achieve cost savings. MUH caretakers provide a unique perspective for understanding the implications of smoke-free policies because of their role in property maintenance. We examine MUH caretakers’ perceptions regarding the economic and personal impact of smoke-free policies at their properties. Methods: We conducted and analyzed qualitative interviews with 20 multiunit housing caretakers from two large property management companies in the southeastern United States that had implemented smoke-free policies. Results: For non-smoking units, caretakers reported shortened turnover times, in addition to reduction in the need for turnover supplies and capital replacements. Caretakers reported an improvement in their work environments due to reduced workload and exposure to secondhand and thirdhand smoke after implementation of smoke-free policies. Conclusion: The potential for cost savings exists for MUH operators who enact smoke-free policies because of decreased labor, supplies, and capital costs. Smoke-free policies may also improve the work environment of caretakers and other frontline MUH employees. These are important findings for MUH companies seeking to lower their operation costs and improve their employees’ working conditions, as well as for smoke-free advocates seeking to promote policy change.

## 1. Introduction

Secondhand smoke, or the smoke from burning tobacco products or exhaled by a smoker, is known to cause respiratory infections, ear infections, and asthma attacks in children, as well as coronary heart disease, stroke, and lung cancer in non-smoking adults [1]. There is no risk-free level of secondhand smoke exposure [2]. In the United States, an estimated 30 million out of 79 million multiunit housing (MUH) residents who do not smoke are exposed to secondhand smoke in their living units [3]. Thirdhand smoke, or the residual tobacco smoke pollutants left on indoor surfaces after a cigarette is extinguished, also poses health risks [4] including increased cancer risk in non-smokers [5]. One study examining thirdhand smoke exposure found that non-smoking residents who moved into houses, apartments, and condominiums formerly occupied by smokers continued to be exposed to thirdhand smoke two months after moving in [6].

A substantial proportion of MUH residents are low-income [7], and the prevalence of smoking is higher among Americans living below the poverty level [8]. Smoke-free policies have been shown to reduce exposure to secondhand smoke and the incidence of adverse health events among non-smokers [2]. For this reason, the United States Department of Housing and Urban Development (HUD) urges owners and operators of publicly and privately owned subsidized housing to take their properties smoke-free [9,10].

One motivation for MUH owners and operators to implement smoke-free policies is the potential to generate cost savings due to lower turnover costs and fewer smoking-related fires [7]. Few studies, however, have attempted to document the actual cost savings achieved at properties with a smoke-free MUH policy. Ong *et al.* [11] found that properties with a comprehensive smoke-free policy experienced fewer smoking-related costs than properties with a partial or non-existent smoke-free policy, with properties experiencing average smoking-related costs of $282 per unit. Further, in a study of affordable housing properties in North Carolina, property managers reported that turnover costs are on average $348 higher in units where tenants have smoked [12].

While previous research has investigated smoke-free policies in MUH from the perspective of owners, managers, and residents, this paper examines smoke-free policies from the viewpoint of MUH caretakers. “Caretakers” is the term used by the property management companies included in this study to refer to employees who perform maintenance-related tasks and rehabilitate, or turn over, vacated units when residents move out; however, other companies in the MUH industry may use different terms for this position, such as maintenance worker. Caretakers are not to be confused with site managers, who oversee leasing and rule enforcement issues for a property. Caretakers are intimately involved in daily property operations and provide a unique perspective for understanding the implications of smoke-free policies in MUH, including the potential for cost savings.

## 2. Experimental Section

We chose a phenomenological qualitative research design in order to better understand caretakers’ perspectives on the transition to smoke-free housing in two large affordable housing management companies, Landura Management Associates (Landura) and Partnership Property Management (PPM). Both companies are headquartered in North Carolina and manage privately owned, government subsidized properties in multiple southeastern states. Since the properties these companies manage are privately owned they are not considered public housing but are considered affordable housing, since government subsidies reduce rental prices. Landura manages 95 properties in six states and took the majority of its properties smoke-free in 2011 (Personal conversation with Landura executives). PPM manages 263 properties in five states, with all transitioning to smoke-free by 1 January 2014 (Personal conversation with PPM executives). We obtained approval for the study from the Institutional Review Board of the University of North Carolina at Chapel Hill. Landura and PPM executives provided additional consent for the study.

### 2.1. Participants

The study participants were caretakers at properties managed by Landura and PPM. Participant eligibility required an employment start date preceding and continuing employment following the implementation of a smoke-free policy at the caretaker’s property. Landura and PPM executives identified eligible caretakers and provided researchers with caretaker contact information. Researchers contacted participants by telephone to set interview times. In order to reach saturation, or the collection of data until no new information is received [13], researchers conducted interviews with 20 MUH caretakers from North Carolina (*n* = 12), Virginia (*n* = 3), South Carolina (*n* = 2), Tennessee (*n* = 2), and West Virginia (*n* = 1). Ten caretakers were recruited from Landura and 10 were recruited from PPM. Each of the 20 caretakers interviewed managed different properties. Demographic information was not collected for study participants, as it was not considered germane to the study’s main purpose of examining the impacts of smoke-free policies on costs. Caretaker participation in interviews was voluntary and no compensation was offered. One caretaker who had initially agreed to take part in the study was subsequently unable to be reached, and was therefore not included.

### 2.2. Data Collection

Four of the coauthors (*i.e.*, EP, OA, OM, RW), along with an additional staff person, each conducted phone interviews with four MUH caretakers from September through November of 2014, with one Landura interview taking place in July 2014. Caretakers provided verbal consent at the time of the interview. Interviews lasted between 15 and 30 min and were audio recorded. Immediately following each interview, researchers transcribed the audio recordings verbatim.

Researchers conducted interviews using a semi-structured discussion guide developed through formative research and then pilot-tested. First, researchers obtained information on caretakers’ length of employment with the company, the number of properties at which they worked, and typical daily work activities. Following these initial questions, researchers explored issues surrounding turnover of non-smoking and smoking units. For the purposes of this study, we conceptualized non-smoking units as those in which a non-smoker had been the most recent resident. We defined smoking units as those in which a smoker had been the most recent resident. Interview questions pertained to supplies and equipment needed for turnover, contractor use, and time required to prepare a unit for a new resident. After the differences between turnings over a non-smoking *vs.* a smoking unit were established, researchers questioned participants on any smoke and fire damage caused by smoking in units and on the overall impact of smoke-free policies on their jobs.

### 2.3. Data Analysis

Researchers performed analysis using the qualitative analysis software, Dedoose 5.0.11 [14], de-identifying each of the 20 completed transcripts prior to analysis. A codebook was generated, and one transcript was chosen at random for individual coding by two researchers. Researchers discussed differences in coding, agreed on standard code application, and edited the codebook to address ambiguity. A different transcript was chosen, again at random, and coded independently by the same two researchers in order to determine reliability of coding. Once coder reliability was established at 100%, each transcript was coded, with 10% double coding to ensure quality control. Themes and frequency of codes were identified by the research team using Dedoose’s analysis tools.

## 3. Results

The caretakers interviewed had been employed by their respective management companies for an average of 9.9 years, with a range of two to twenty-seven years. They worked at an average of two properties, with the range extending from one to five. Typical daily activities for caretakers included cleaning of the property and grounds, as well as maintenance including painting, landscaping, and general repairs. Qualitative analysis revealed several key themes that highlighted caretakers’ perspectives on the smoke-free policy change. These included: (1) decrease in time needed for unit turnover; (2) decrease in supplies needed for unit turnover; (3) decrease in capital replacements necessitated by smoke damage; and (4) positive caretaker experiences with smoke-free policies.

### 3.1. Decrease in Time Needed for Unit Turnover

Every caretaker reported that turning over a non-smoking unit takes less time than turning over a smoking unit, with many indicating that non-smoking units take half the amount of time to turn over as smoking units. Repeatedly, caretakers expressed that time differences resulted from the fact that smoking units took longer to clean and to paint: 

*Less work cause there is less painting, which means more time to do other things··· the painting is the biggest issue, less painting, less cleaning of the appliances because, you know, of course nicotine gets on everything so it does cut down on my time and turning over the unit.*

*Cleaning wise, it takes a little extra [time] because… it has that tar on it. And of course it takes a little extra time for a cleaner to clean windows, the cabinets and, depending on how bad the smoking unit is, it takes a little extra time to clean the bathrooms and the vents.*

*Some of the ones that were really heavy smokers that we had to clean, it’s taken up to 8 h to clean one with two people. Versus a [non-smoking] unit you might have it done in four hours. There’s a big difference in the cleaning part.*

*So normally we’ll spend about $85 [to pay contractors] to clean the apartment. With a smoker, they have to get the nicotine off everything, racks, doors, blindfolds, tubs, everything else that cost me pretty close to $400 to do that. The painting, they have to come in and put a white shellac over the walls because of the nicotine stains, you can smell it if you do not do that. So you essentially take an apartment that would cost you $350 is going to run you $700 [in contractor costs] because you are essentially painting it twice.*

*If we had one that was very heavily soiled with nicotine, it involved pretty deep washing of the walls and windows, prior to painting and cleaning. In other words, it wouldn’t be uncommon to have a wall that was just streaked with nicotine. And same thing on the glass, if somebody just really smoked a lot, all of the light, light fixtures were coated in nicotine. The air conditioning coils and filters had nicotine on em. Just, the ceilings were, were dingy and just about all of that is gone now, which is wonderful for me.*

Some caretakers indicated that while their overall workload was reduced as a result of the smoke-free policy, there was a small spillover effect as smokers moved their activities outdoors: 

*[Residents are] supposed to go outside to smoke, they’re supposed to go 25 feet from the building. That has created a little problem in some places where some people they throw the butts on the ground. So that’s extra work that we have to do, that I have to pick up.*

*It’s not a huge amount, but yeah, I’d say cigarette butts have increased outside. I sure think they have.*

One MUH site deterred this negative behavior through an additional policy: 

*For a while, I did have to pick up more butts; but that’s not the case anymore. If we ride by and see that, we can charge them $25…. For the first month, we saw that situation and then once we told them about the fine, they stopped.*

### 3.2. Decrease in Supplies Needed for Unit Turnover

Caretakers described the type and quantity of supplies they used when turning over non-smoking and smoking units. They reported using a variety of supplies to remove smoke damage and nicotine stains in smoking units, including primers, degreasers, stain blockers, odor eliminators, bleach, and all-purpose cleaners: 

*Depends on how bad the smoker is…we buy this degreaser and it’s a powerful clean up, we spray it down and it runs the tobacco off of it.*

*Usually what I do, I would put some ZEP commercial and fabric odor eliminator. I would actually put that in my shampooer and go over the carpet a couple times with it. Sometimes if it’s really, really bad, I would have to sort of leave the windows open to air the apartment out.*

*Generally you can mix that up with hot water and 409 [All-Purpose Cleaner], personally I don’t do it···but I’ve been to some complexes where they use Clorox. Basically a sponge-mop and hot water and 409 will remove all nicotine from the walls.*

Overall, caretakers indicated that they used supplies—specifically, paint and primer—in larger quantities when turning over a smoking unit compared to a non-smoking unit: 

*You use the same [supplies] in both, but you use more…. Most times, you don’t have to use a primer in an apartment, but on a smoker, you have to use primer because if you don’t, it [nicotine] bleeds back through.*

*I [add Kilz brand primer to] all the walls and then do a repaint and sometimes I’ve had to put two coats of Kilz on there to cover the smell.*

*If you have a heavy smoker, the nicotine just sticks to the wall…it’s several shades darker with nicotine. And without several coats of paint on it, it would bleed through so you would have to paint it several times.*

### 3.3. Decrease in Capital Replacements Necessitated by Smoke Damage

An important theme that emerged was the extensive capital replacements caused by smoke damage, including damage from smoking-related fires. The unit features that require more frequent replacement in smoking units included carpets, blinds, ventilation systems, and kitchen and bathroom appliances, resulting in potentially higher capital costs for turning over smoking units: 

*We probably keep carpets for 5 to 6 years. If you have a smoker, you’re lucky if you get 1 to 2 years out of the carpet and if you’re going to re-rent it, it’s mandatory that it has to be replaced. Also, the cleaning cost jumps up. [Costs] go [up] about three to four times to clean.*

*I will say that it takes a lot to get the smell out of the vents. If you smoke four packs a day for eight years in the apartment, it is very difficult to clean that up. It turns everything yellow: Refrigerator, stoves, cabinets, you got to address all those issues. You’ve got to replace the stove and the refrigerator because they will not come clean it, you know, so much smoke. 9 times out of 10 you will have to replace the mini blinds in all heavy smokers’ units.*

*They would leave [cigarettes] there, you know, on the countertops, sometimes you would have to replace a counter.*

*[In units where someone smoked] you gotta replace everything. I mean, we’re talking not only carpet but also all the smoke detectors, everything, because everything is just destroyed, light fixtures, everything.*

*I would lose maybe $200 worth of thermostats per year because they were just so smoky and dingy.*

*I’ve had to change range hoods because [cigarette smoke] gets up into the filters of that, your air conditioning units you’ve had to treat the duct works because of nicotine.*

Some caretakers brought up the consequences of fire damage resulting from smoking within units. There was one particular incident that caused extensive damage to an apartment building, resulting in the replacement of ten units: 

*We had a woman that went to sleep smoking with oxygen and it burnt ten apartments···It was like a million dollar damage. They tore the ten apartments down and rebuilt em…. The whole apartment complex, all the 40 residents had to be moved out for a month because we had to get all the water and stuff out, because the water had run through…we had to get all the smoke out of the apartments. We had to fix the carpets in the halls where the fireman had drug the lines down, they had to replace carpet in the other ones where water had run into apartments. It was major.*

### 3.4. Positive Caretaker Experiences with Smoke-Free Policies

Many caretakers indicated that the adoption of the smoke-free policy had improved their ability to do their job. In addition to unit turnover becoming easier, the policy reduced adverse effects on their health and increased satisfaction working in a smoke-free environment. There were some reports of negative health effects resulting from working in MUH prior to the implementation of the smoke-free policy: 

*I’m very grateful that I don’t have to breathe in that secondhand smoke when I walk into an apartment. I think at times it created allergy problems…. There was a tremendous amount of secondhand smoke in the apartments when I walked in.*

*Yeah, it’s nice for me to go into an apartment and not smell smoke, because I’m very cognitive of smoke, and it can get very overwhelming if you have to go in there and work on the apartment. So my own personal health yes, it is much nicer without the smoke.*

Aside from health concerns, there was a reluctance to work in smoking units stemming from individual preferences. This was revealed by caretakers who mentioned that they were non-smokers themselves: 

*When we were [working in] a smoking building, I asked the manager to request that··· [the residents] were not smoking while we were there, however long we were gonna be there, 15 to 20 minutes or so… but when we would go in there, it bothered me because I’d come out smelling like an ashtray…*

*I know that before [the policy], and I’m a non-smoker myself, and when I go in the ones that people smoke in, it bothers me and I just have to do what I have to do.*

*And that’s my opinion [about the smoke-free policy], I mean, I like it. It don’t stink as bad.*

One caretaker in particular discussed smoke-free policies in MUH as part of a larger overall trend towards smoke-free policies in public spaces. He also raised the issue of discrepancies in the existence of smoke-free policies across the spectrum of workplace environments. 

*I would very much like to see us go to a smoke-free campus. I think that given the fact that we’re [government] funded here and…basically everybody here is getting a significant subsidy on their rents, that there’s certain rules that should go with that. And I think one of them should be basically to eliminate smoking completely…. People are used to [smoke-free policies] at hospitals and colleges. And I’m sure your offices are smoke-free…. I think as a society we need to work that way.*

While the general consensus among caretakers was that smoke-free policies were beneficial at their properties, one caretaker questioned the priorities set forth by the policy: 

*Being a smoker, I might be a little biased but I think it’s unfair to say you can’t smoke, giving more rights to non-smokers than to smokers. There’s kind of an issue with… people [who] are using drugs. Isn’t that a bigger threat than a smoker? … My personal stance is that this apartment complex was built in 1983. There’s been 30 years of smoking in it and then all of a sudden transitioning it to non-smoking seems a little odd because you still have nicotine residue in the walls.*

## 4. Discussion

To our knowledge, no previous studies have addressed MUH caretakers’ perspectives on smoke-free policies. Caretakers’ roles in day-to-day property operations allow them special insight into the impact of smoke-free policies, including cost savings.

The key finding of this study was that caretakers perceived a notable decrease in the time needed to turn over non-smoking units compared to smoking units. As smoke-free policies eliminate this latter category of units, smoke-free policies may translate to significantly lower labor costs for turnover of units. While not examined in this study, reduced turnover times may also allow managers to fill units with new residents more quickly, resulting in increased income for the property. Caretakers also reported a decrease in the quantity of supplies necessary to turn over non-smoking units. This was primarily due to the need for fewer coats of primer and paint and a decrease in the amount of cleaning supplies used. There was also a reduction in replacement of features in non-smoking units, including ventilation systems, kitchen and bathroom appliances, and carpets. A final way in which smoke-free policies may contribute to cost savings is in the prevention of fires [7]. One caretaker in this study described a fire at his property that was financially devastating. From 2007–2011, fires caused by smoking materials created an annual average of $215 million in damages in MUH properties in the United States [15]. Although no study has yet demonstrated a decrease in fires at properties with smoke-free policies, one may hypothesize that smoke-free policies may decrease the amount of smoking in apartments and thus the number of fires caused by smoking materials. This study, therefore, highlights several mechanisms—labor, supplies, and capital costs—by which smoke-free policies may result in cost savings for MUH operators. While previous studies show that MUH operators report cost savings at smoke-free properties [11,12], this study fills gaps in knowledge about what factors may contribute to these cost savings.

An additional finding of this study was that caretakers perceived smoke-free policies in MUH to be beneficial to their jobs. This was the result of reduced time in turning over non-smoking units compared to smoking units and perceived health benefits from working in a smoke-free environment. While caretakers did not use the term “thirdhand smoke” when discussing health concerns, exposure to residual smoke in units was clearly a concern. The levels of tobacco smoke exposure and subsequent health impacts among MUH caretakers have not been studied but have been well documented among other populations commonly exposed to smoke in the workplace, such as hospitality workers [16,17,18]. Just as policymakers have passed smoke-free policies in bars and restaurants to protect workers from smoke exposure on the job, owners of smoke-free housing may be moved to implement smoke-free policies by evidence that their caretakers and other on-site workers are at risk from smoke exposure. Further research is needed to examine the impacts of secondhand and thirdhand smoke exposure on MUH employee health.

While smoke-free policies may lead to cost savings and reduced smoke exposure among employees in MUH, smoke-free policies are still not the norm in MUH. A 2013 survey of affordable housing properties in North Carolina found that 16.5% of properties had all smoke-free units [19], while studies in other parts of the country (Western New York state; Douglas County, Nebraska; and four cities in Virginia) showed that 9%–16% of properties had all smoke-free units [20,21,22]. In 2012, 18.1% of American adults smoked, with higher rates among persons with low incomes [23] and persons living in the southeastern United States [24]. As such, smoke-free policies could prevent a substantial amount of smoking in the MUH setting, particularly in affordable housing in the southeastern United States.

This study has several limitations, including the inability to definitively attribute cost savings to smoke-free policy change using qualitative methods. We initiated this research as a mixed-methods study and attempted to examine actual costs savings for Landura and PPM at the property level. In the quantitative study (not reported here), we examined budget categories such as labor and maintenance, repairs and capital replacements, paint, and annual capital budget. However, we found during analysis that individual property savings were reinvested into property operations, making it impossible to decipher true cost savings related to the smoke-free policy. Other studies have examined the differences in turnover costs by relying on respondents’ estimates of costs rather than actual cost records [11,12]; these estimates may be subject to recall bias and cannot be easily independently verified. Future research should examine whether there are methods to capture the cost savings from smoke-free policies in methodologically rigorous ways.

An additional limitation is the fact that the caretakers interviewed were based in southeastern states, with the majority being located in North Carolina. Our findings may not be generalizable to other regions. Furthermore, this study took place in the affordable housing arena and our findings may not be generalizable to market-rate properties. Finally, while caretaker interviews took place several years after smoke-free policy implementation at Landura, they took place less than a year after policy implementation at PPM. Arguably, PPM caretakers had a limited window in which to observe the effects of smoke-free policy implementation. Given, however, the extended period of time for Landura caretakers to observe the policy changes and the similarity in findings between the two management companies as well as the fact that responses drew upon PPM caretakers’ years of experience turning over smoking units under the old policy, we believe that the implications of this limitation are likely small.

## 5. Conclusions

Caretakers reported significant reductions in turnover time, supplies, and capital replacements for non-smoking units. These findings have the potential to lead to savings in labor, supplies, and capital costs for MUH companies that implement smoke-free policies. In addition, smoke-free policies in MUH have garnered the support of caretakers due to the policies’ contribution to decreased workload and improved work environment and occupational health. These are important findings for MUH companies looking to lower operation costs, as well as for smoke-free advocates seeking to promote policy change.
